# Evaluation of a fentanyl drug checking service for clients of a supervised injection facility, Vancouver, Canada

**DOI:** 10.1186/s12954-018-0252-8

**Published:** 2018-09-10

**Authors:** Mohammad Karamouzian, Carolyn Dohoo, Sara Forsting, Ryan McNeil, Thomas Kerr, Mark Lysyshyn

**Affiliations:** 1 British Columbia Centre for Excellence in HIV/AIDS, St. Paul’s Hospital, 608-1081 Burrard Street, Vancouver, BC V6Z 1Y6 Canada; 2 School of Population and Public Health, University of British Columbia, 5804 Fairview Avenue, Vancouver, BC V6T 1Z3 Canada; 3 HIV/STI Surveillance Research Center, and WHO Collaborating Center for HIV Surveillance, Institute for Futures Studies in Health, Kerman University of Medical Sciences, Kerman, 7616913555 Iran; 4 Public Health Agency of Canada, Ottawa, ON K1A 0K9 Canada; 5 Vancouver Coastal Health Authority, Vancouver, BC V5Z 4C2 Canada; 6 Department of Medicine, University of British Columbia, St. Paul’s Hospital, 608-1081 Burrard Street, Vancouver, BC V6Z 1Y6 Canada

**Keywords:** Drug checking, Substance use, Injection drug use, Supervised injection facility, Canada

## Abstract

**Background:**

British Columbia, Canada, is experiencing a public health emergency related to opioid overdoses driven by consumption of street drugs contaminated with illicitly manufactured fentanyl. This cross-sectional study evaluates a drug checking intervention for the clients of a supervised injection facility (SIF) in Vancouver.

**Methods:**

Insite is a facility offering supervised injection services in Vancouver’s Downtown East Side, a community with high levels of injection drug use and associated harms, including overdose deaths. During July 7, 2016, to June 21, 2017, Insite clients were offered an opportunity to check their drugs for fentanyl using a test strip designed to test urine for fentanyl. Results of the drug check were recorded along with information including the substance checked, whether the client intended to dispose of the drug or reduce the dose and whether they experienced an overdose. Logistic regression models were constructed to assess the associations between drug checking results and dose reduction or drug disposal. Crude odds ratios (OR) and 95% confidence intervals (CI) were reported.

**Results:**

About 1% of the visits to Insite during the study resulted in a drug check. Out of 1411 drug checks conducted by clients, 1121 (79.8%) were positive for fentanyl. Although most tests were conducted post-consumption, following a positive pre-consumption drug check, 36.3% (*n* = 142) of participants reported planning to reduce their drug dose while only 11.4% (*n* = 50) planned to dispose of their drug. While the odds of intended dose reduction among those with a positive drug check was significantly higher than those with a negative result (OR = 9.36; 95% CI 4.25–20.65), no association was observed between drug check results and intended drug disposal (OR = 1.60; 95% CI 0.79–3.26). Among all participants, intended dose reduction was associated with significantly lower odds of overdose (OR = 0.41; 95% CI 0.18–0.89).

**Conclusions:**

Although only a small proportion of visits resulted in a drug check, a high proportion (~ 80%) of the drugs checked were contaminated with fentanyl. Drug checking at harm reduction facilities such as SIFs might be a feasible intervention that could contribute to preventing overdoses in the context of the current overdose emergency.

## Background

Among the most alarming drug trends in North America is the rapidly increasing impact of illicit drugs adulterated with illicitly manufactured fentanyl [1, 2]. While fentanyl can be prescribed to treat pain, it has high toxicity relative to morphine or heroin and is considerably more likely to result in a fatal overdose, a high potency that has drastically changed the substance use landscape in North America [1, 3–6]. In the USA, the Centers for Disease Control and Prevention reports that fentanyl was detected in 56.3% of 5152 opioid-related overdose deaths across 10 states in the second half of 2016 [7].

Canada is facing a similar unprecedented opioid-related overdose epidemic, where exposure to illicit fentanyl can come from a number of sources including counterfeit opioid tablets (e.g. fake oxys) [5], heroin contaminated with fentanyl [1, 5], fentanyl patches from either illicit or pharmaceutical sources [1], and stimulants such as cocaine contaminated with fentanyl [2, 8]. British Columbia (BC) is one of the settings that have witnessed a sharp increase in the rate of opioid-related overdose deaths leading to a public health emergency declaration in April 2016 [1, 2, 5]. The surge in the number of fentanyl-detected overdose deaths in 2017 among people who use drugs (PWUD) in BC is very concerning; 999 fentanyl-detected overdose deaths were identified from January to October 2017 compared to 654 in 2016, 151 in 2015, and 91 in 2014 [9].

Exposure to illicitly manufactured fentanyl may be unintentional [10], and PWUD may be unaware of fentanyl presence in their drugs. For example, among 231 patients undergoing opioid withdrawal management in Massachusetts, two thirds of those who reported never being intentionally or unintentionally exposed to fentanyl, tested positive for fentanyl [11]. Furthermore, a recent survey of 242 clients of 17 harm reduction sites in BC detected fentanyl in 29% of the participants, 73% of whom were not knowingly using fentanyl [10]. On the other hand, given the ongoing overdose epidemic, many PWUD may suspect their drugs to be adulterated with fentanyl; however, they have no reliable way of knowing which drugs are adulterated before they use them.

In response to the overdose crisis in BC, interventions are now being implemented and scaled up, including the piloting of fentanyl drug checking services at Insite (i.e. a supervised injection facility [SIF] which provides a hygienic environment where individuals can inject their drugs under the supervision of qualified staff) [12–14]. Drug checking is a harm reduction intervention that has been implemented in a variety of settings. It was introduced in Europe following the establishment of the Drug Information and Monitoring System in the Netherlands in 1992 (i.e. a national system of stationary testing facilities across various regional institutes catered towards substance use prevention and care). Drug-checking offers testing of street drugs to assess their composition (including potential contaminants) and allows for more informed decision-making by PWUD [15–17]. Drug-checking services can vary in a number of ways including testing method (e.g. colorimetric reagents, high-performance liquid chromatography, gas chromatography, mass spectrometry), type of results available (e.g. presence or absence of a component, quantitative information about all compounds), setting (e.g. at home, mobile, remote site), and purpose (e.g. individual harm reduction, public health action, market monitoring) [18]. While drug checking services have been shown to be effective in reaching young people who use drugs for recreational purposes and persuading them to change their behaviour positively [19], they have also been criticized for creating an unjustified feeling of safety about illicit drugs while the absence of unexpected or potent components in a sample of illicit drugs cannot guarantee its safety [18, 20].

While drug checking services have been available across numerous European countries such as the Netherlands, France, Austria, Belgium, Portugal, Spain, and Switzerland for over two decades [16–18, 21, 22], they are considered illegal and thus remain underdeveloped in Canada even though illegal drug checking services have been implemented at some music festivals [9, 23]. The goal of the drug checking service that has been operating at Insite since July 2016 is to improve clients’ awareness of their exposure to fentanyl and improve our understanding of the drug supply. Improved awareness of fentanyl exposure may encourage client adoption of available harm reduction practices. Therefore, this study aims to evaluate the drug checking service using data collected at Insite. In particular, this study assesses the fentanyl drug checking positivity rate, the prevalence of fentanyl contamination by substance type, as well as the impact of fentanyl drug checking results on intention to reduce their dose or dispose of their drug, overdose, and the need for naloxone administration. Given the clear and urgent need for novel interventions to address the overdose epidemic and limited body of evidence on the evaluation of drug checking services, the findings of this study have potential to inform current overdose prevention and harm reduction efforts.

## Methods

### Setting

Insite is North America’s first government sanctioned SIF that offers supervised injection services in Vancouver’s Downtown East Side, a neighbourhood with high levels of injection drug use and related harms, including overdose deaths [12, 13]. Insite aims to reduce harms to PWUD’s health while linking them to care and treatment [12, 13]. Insite operates under an exemption to Canada’s Controlled Drugs and Substances Act which allows clients to possess and use drugs on site and which permits the drug checking service to operate in a legal manner in this setting [12, 13]. Since its establishment in 2003, there have been over 3.6 million visits to the facility, and over 6000 overdoses have been treated, none of which has been fatal. Insite clients are mostly high-intensity injection drug users who often come from an extremely marginalized background (e.g. unstable housing) [24, 25].

### Data collection

**Fig. 1:**
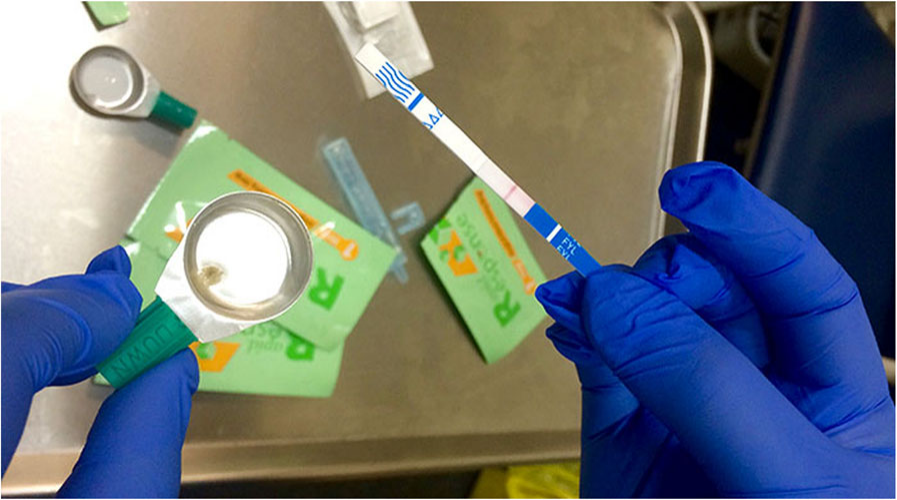
A test strip used to check drug samples for the presence of fentanyl at Insite. (Image provided by Vancouver Coastal Health)

Insite’s clients were notified of the availability of drug checking service at Insite through posters set up at the facility. Insite’s staff offered all clients the opportunity to check their drugs for the presence of fentanyl as they entered the injection room by asking ‘Do you want to check your drugs for fentanyl?’ Consenting participants were then instructed to dissolve a small drug sample (i.e. the size of a grain of salt) in water in a cooker and then test it with a BTNX Rapid Response Fentanyl Test Strip prior to consumption. These strips—which are not designed to test drugs at SIFs—utilize an enzyme immunoassay test which uses an antibody’s bonding with an antigen to signal the presence of fentanyl qualitatively (i.e. presence vs. absence) and are inexpensive (1$ each), simple to use, and easy to read [26]. Moreover, BTNX strips have a detection limit (i.e. the lowest concentration that could be detected) of 0.13 μg/ml and have been shown to be highly sensitive and specific when used in this way [26]. Fentanyl drug checks could be performed before or after drug consumption, depending on the clients’ preference. Post-consumption checks were done using drugs that had not been consumed or residue left in the cooker that was used to prepare the drugs (Fig. 1). Once the result of the test was confirmed by Insite staff, they were recorded on the reporting form as either ‘Positive’ or ‘Negative’ and whether they tested pre- or post-consumption along with the following information: client-reported substance (e.g. heroin or methamphetamine or cocaine); substance dose reduction intention (yes or no); substance disposal intention (yes or no); overdose following consumption (yes or no); and naloxone administration among those who overdosed (yes or no). Overdose was determined on site by Insite nursing staff, and naloxone was administered according to Insite clinical protocols. Given the anonymous nature of data collection, no demographic or identifying information was collected and participants’ unique identification codes (i.e. Insite ID) were not linked to the study data.

Participants were notified of the result of the test. If the test result was negative, participants were informed that the test strip only tested for fentanyl and their negative test result could not ensure that their drugs were not adulterated with other substances which could be more potent than fentanyl (e.g. W-18). Participants were also asked if they planned to reduce their dose or dispose of their drugs. Finally, participants were offered any or all of the following interventions had they not been accessing them already: information on reducing harm from injection (e.g. use a little, do not use alone); Take Home Naloxone and training; information on availability of other SIF; and offer to connect with addiction treatment services (e.g. Detox/Daytox, addiction counselling, opioid substitution therapy).

### Data analysis

In this cross-sectional study, we used data from the fentanyl drug checking service at Insite collected from July 7, 2016, to June 21, 2017. Eligible participants for the analysis included any individual who had accessed the fentanyl drug checking programme at Insite during the study period. Frequencies and descriptive statistics were computed for all variables. Fentanyl drug checking positivity percentage within Insite was calculated using the following formula: (number of positive checks/total number of checks) × 100.

Unadjusted bivariable logistic regression models were constructed to investigate the associations between drug checking results and intentions for dose reduction or drug disposal. Logistic regression models were also used to assess the associations between drug checking results (pre- or post-consumption) and dose reduction intentions with overdose events as well as naloxone administration. Crude odds ratios (OR), as well as 95% confidence intervals (CI), were reported. Stata version 14 (Stata Corp.) was used throughout the analysis, and *P* values less than 0.05 were considered statistically significant.

### Ethics approval and consent to participate

This study involved the secondary use of anonymous data collected as part of the drug checking service. Participants’ refusal to participate in the study did not influence the services provided to them. The University of British Columbia (UBC) and Providence Health Care ethics committees reviewed and approved the study protocol (UBC-REB NUMBER: H16-02973).

## Results

From July 7, 2016, to June 21, 2017, there was a total of 134,176 visits to Insite with an average of 533 daily visits (range 387–780). Since implementing the fentanyl drug checking programme, a total of 1411 (1%) visits led to a drug check conducted by Insite clients. This represents a daily average of 4.0 checks with a range of 0 to 27 checks per day. Out of all drug checks performed during the study period, 1121 (79.8%) were positive for fentanyl. The majority of drug checks were performed on client-reported heroin, 84.1% (*n* = 939) of which tested positive for fentanyl. The majority of checks were performed post-consumption (58%; *n* = 789). Drugs checked post-consumption were significantly more likely to be positive for fentanyl compared to those checked pre-consumption (82.9%; *n* = 654 vs. 76.5%; *n* = 438; *P* value 004).

Among those with a positive drug check pre-consumption, 36.3% (*n* = 142) reported planning to reduce their drug dose, and the odds of dose reduction intention among those who had a positive drug check were significantly higher than those with a negative result (OR = 9.36; 95% CI 4.25–20.65). Conversely, among those with a positive drug check pre-consumption, only 11.4% (*n* = 50) planned to dispose their drug. Although the odds of drug disposal intention were higher when the drug check was positive, the association was not statistically significant (OR = 1.60; 95% CI 0.79–3.26). Detailed association of drug checking and intentions for dose reduction or drug disposal are presented in Table 1.

**Table 1:** Association of drug check results and intentions for dose reduction or drug disposal of Insite clients who used a fentanyl drug checking service in Vancouver, Canada

Drug check result^a^	Total	Dose reduction Yes; *n* (%)^b^	Dose reduction No; *n* (%)	Odds ratio (95% CI)	*P* value^c^
Positive	391	142 (36.32)	249 (63.68)	9.36 (4.25–20.65)	0.0001
Negative	122	7 (5.74)	115 (94.26)	Ref.	
Drug check result^a^	Total	Drug disposal Yes; *n* (%)	Drug disposal No; *n* (%)	Odds ratio (95% CI)	*P* value^c^
Positive	436	50 (11.47)	386 (88.53)	1.60 (0.79–3.26)	0.186
Negative	134	10 (7.46)	124 (92.54)	Ref.	

^a^Limited to pre-consumption checks. ^b^All percentages are row percentage. ^c^*P* values based on chi-square and Fisher’s exact test as appropriate

During the study period, Insite’s staff reported a total of 120 overdoses in association with drug checks; most of which (94%; *n* = 113) were reported among those who tested post-consumption. The odds of overdose among those who had a positive drug check were significantly higher than those with a negative drug check (OR = 5.97; 95% CI 2.41–14.78). Of the total recorded overdoses, 76.2% (*n* = 92) required naloxone administration and the odds of naloxone administration among those who had a positive drug check were significantly higher than those with a negative drug check (OR = 4.42; 95% CI 1.77–11.02). Moreover, of those who planned to reduce their dose, only 4.5% (*n* = 7) overdosed and 3.2% (*n* = 5) were administered naloxone. Among all participants, dose reduction intention was significantly associated with lower odds of overdose (OR = 0.41; 95% CI 0.18–0.89) and naloxone administration (OR = 0.38; 95% CI 0.15–0.96). Detailed statistics on the association of drug check results and overdose as well as naloxone administration are presented in Table 2.

**Table 2:** Association of drug check results and overdose as well as naloxone administration among clients of Insite who used a fentanyl drug checking service in Vancouver, Canada

Drug check result	Total	Overdose Yes; *n* (%)^a^	Overdose No; *n* (%)	Odds ratio (95% CI)	*P* value^b^
Overall					
Positive	1028	115 (11.19)	913 (88.81)	5.97 (2.41–14.78)	0.0001
Negative	242	5 (2.07)	237 (97.93)	Ref.	
Pre-consumption					
Positive	357	7 (1.96)	350 (98.04)	4.60 (0.26–81.21)^c^	0.297
Negative	107	0 (0.00)	107 (100.00)	Ref.	
Post-consumption					
Positive	649	108 (16.64)	541 (83.36)	4.95 (1.97–12.39)	0.0001
Negative	129	5 (3.88)	124 (96.12)	Ref.	
Drug check result	Total	Naloxone administered Yes; *n* (%)	Naloxone administered No; *n* (%)	Odds ratio (95% CI)	*P* value^b^
Overall					
Positive	1026	87 (8.48)	939 (91.52)	4.42 (1.77–11.02)	0.001
Negative	244	5 (2.05)	239 (97.95)	Ref.	
Pre-consumption					
Positive	355	3 (0.85)	352 (99.15)	1.83 (0.09–35.87)^c^	0.688
Negative	109	0 (0.00)	92 (100.00)	Ref.	
Post-consumption					
Positive	649	84 (12.94)	565 (87.06)	3.68 (1.46–9.27)	0.003
Negative	129	5 (3.88)	124 (96.12)	Ref.	

^a^All percentages are row percentage. ^b^*P* values based on chi-square and Fisher’s exact test as appropriate. ^c^As zeros caused problems with computation of the odds ratio or its confidence interval 0.5 added to all cells [36]

## Discussion

Our study revealed that only a small proportion of drugs used at Insite during the study period were checked using the drug checking service. However, a high proportion (~ 80%) of the drugs checked was found to be contaminated with fentanyl. We also observed that PWUD who received a positive drug check pre-consumption were significantly more likely to plan to reduce their drug dose upon injecting but not more likely to plan to dispose of their drugs. Our results are comparable with Health Canada’s Drug Analysis Service laboratory reports that suggests a high and increasing proportion of illicit drugs seized by law enforcement agencies in BC were contaminated with fentanyl during this period [27]. However, they are considerably higher than the positivity rates in previous assessments in BC including studies that used urine drug screening tests and found that one in three PWUD across 17 harm reduction sites across BC [10] and one in six PWUD in Vancouver [28] tested positive for fentanyl. While our findings are specific to drugs checked at Insite during the study period, they may inform efforts aimed at monitoring and reducing risks in the local drug supply in jurisdictions across North America that are experiencing increased rates of overdose with illicit fentanyl [4, 6, 29].

Drug checking has mostly been implemented at music festivals and in other community settings [17, 22]. While previous studies have analysed the residual content of used syringes in syringe-exchange facilities [30], we believe our study is the first of its kind to examine a legal government-sanctioned drug checking service at a SIF, which is a unique setting for studying drug checking services as it allows for observing clients as they perform drug checking, monitoring adopted harm reduction practices, and documenting relevant health outcomes (e.g. overdose). For instance, in our study, drug checking results encouraged clients to plan to reduce their dose but most did not plan to dispose of their drugs altogether. These findings are different from documented drug disposal practices in music festivals. For example, in 2015 at Shambhala Music Festival—an event in BC with a long history of offering drug checking services—13% of clients disposed of their drugs following an unexpected result compared to 2% following expected results [23]. At Insite, clients may not have planned to dispose of contaminated drugs because they are more likely to be dependent on the drugs they are using, lack funds to purchase replacement drugs, and have no access to unadulterated street drugs. Furthermore, while a recent study has found no evidence of compensatory drug use following naloxone training among a group of heroin users [31], it is possible that some clients may have specifically sought out fentanyl knowing they can be treated for an overdose at a SIF, an assumption that needs to be further explored in our future studies at Insite.

Not surprisingly, a positive drug check result was associated with significantly greater odds of the client experiencing an overdose and requiring naloxone administration. These findings are comparable with a study in a SIF in Sydney, Australia, where fentanyl injections had 4.6 times the risk of resulting in overdose compared to heroin or other prescription opioids combined [32]. Nonetheless, interpretations around our findings of the association of drug check results and odds of overdose should be made with caution as contrary to our expectations; the majority of drug checks in our study were performed post-consumption. While PWUD could choose to have their drugs checked prior to or after consumption, details of the timeline and sequence of decision making to participate in the intervention were not captured. In other words, the motivation to perform a drug check might have been the result of an overdose if PWUD asked for a testing strip after consumption. Conversely, motivation to perform a drug check may have *not* been the result of an overdose if PWUD requested a testing strip before consumption but decided to check their drug post-consumption. While performing a drug check after consumption does not provide the client with an opportunity to reduce their dose or dispose of their drugs before consuming, it might still provide them with valuable information. For example, the drug checking result may help explain why an overdose occurred and may help the client decide how to use drugs still in their possession. A positive result post-consumption might also encourage clients to return to the SIF to consume their next dose. Supervised injection services have been shown to prevent death due to overdose across numerous settings [23]. This study shows that offering drug checking at a SIF might extend their benefits by enabling clients to reduce their risk of experiencing an overdose in the first place. Further research is needed to confirm such effects. It is unclear whether drug checking might have similar impacts in settings where supervised injection services are not available. It should also be noted that as only a small proportion of people accessing Insite utilized the drug checking service, the service might have attracted clients more likely to engage in harm reduction strategies.

Overall, few visits to Insite (1%) during the study period resulted in a drug check. It is difficult to compare this uptake to other settings where drug checking has been implemented. For instance, in 2015 at Shambhala Music Festival, 3224 drug checks were performed during the 5-day festival which involved over 67,000 attendees [23]. The low uptake of the drug checking service can also be compared with the findings of a small survey on a convenient sample of 180 PWUD in the mid-sized city of London in Ontario, Canada, in 2016 where 43% of the participants reported that if provided with the service, they would frequently check their drugs at a SIF [33]. Moreover, in a study of 93 young PWUD in Rhode Island, USA, over 90% of the participants showed a willingness to use take-home rapid fentanyl test strips [11]. These differences which should be interpreted with an eye to the small sample size and social desirability bias of the survey results in London and Rhode Island highlight the need for further research on whether willingness to use drug checking services at harm reduction facilities could predict future service uptake [11, 33]. There also remains a need for complementary qualitative research to examine how the degree of suspected contamination of the street drug supply, other social-structural factors (e.g. drug law enforcement, poverty), or providing peer-led distribution of drug checking services influence drug checking behaviours. It is possible that the limited uptake of this intervention might reflect clients’ reluctance to check their drugs when they suspect the majority of street drugs available to be adulterated [33]. Nonetheless, because up-to-date drug checking results from this study were regularly communicated to clients via posters, it is also possible that even clients that did not perform drug checks themselves might have benefited from results of the drug checking service.

It is also worth noting that further research is needed to understand the limitations of current drug checking technologies including the fentanyl test strips. Previous studies including a recent report by Health Canada have raised concerns about the validity of these test strips in detecting novel analogues of fentanyl in street drug samples and the small possibility for false-negative test results [34], and advocated for employing alternative drug checking technologies with better discriminative abilities (e.g. infrared spectrometry methods) [17]. Moreover, these studies argue that the qualitative detection of fentanyl in drug samples might be of limited value to PWUD, particularly in areas such as Vancouver where fentanyl is being increasingly found in the drug supply [27]. However, the findings of the recent Fentanyl Overdose Reduction Checking Analysis Study (FORECAST) that compared the ability of three drug checking technologies (i.e. BTNX fentanyl testing strips, TruNarc machine, and Bruker Alpha machine) in detecting fentanyl in street drug samples with a gold standard test (i.e. gas chromatograph/mass spectrometer) concluded that the fentanyl testing strips used in this study had the lowest detection limit and the highest specificity and sensitivity for fentanyl among the assessed technologies [26]. Furthermore, fentanyl test strips are considerably cheaper and require minimal training for proper use compared to other testing approaches and therefore seem to be a practical and feasible intervention with a significant potential for reducing harm in the context of the current opioid crisis [26]. Nonetheless, further research is warranted to develop and identify portable and easy-to-use testing technologies capable of detecting fentanyl and its analogues in drug samples in a variety of settings [26]. Moreover, future decisions regarding the provision of drug checking services should consider distributing information alongside the tests about the potential limitations of these technologies and the importance of continuing to use other harm reduction practices and programmes even after using a drug checking service [34].

We would like to acknowledge the limitations of our study. The test strips used were not designed to check drug samples in a SIF. However, the findings of the FORECAST study suggest that these test strips are relatively accurate in detecting fentanyl in street drugs samples. Moreover, the anonymous nature of our data restricted our analysis. It was impossible to interpret the findings per individual clients; it is not clear how many individual clients made use of the drug checking service or whether clients who used the service continued using it regularly. Moreover, we may have collected data from clients more likely to have fentanyl present in their drugs. Given the limited generalizability of our findings, future research should seek to combine drug checking data with client SIF utilization data to generate more detailed analysis specific to this issue.

## Conclusions

This study suggests that a high portion of illicit drugs checked at Insite might be adulterated with fentanyl. While responding to the overdose epidemic requires a multifaceted approach [29, 35], drug checking might be an additional harm reduction strategy that could contribute to preventing overdoses in the context of a street drug supply contaminated with illicit fentanyl. This study shows that it may be feasible and potentially useful to offer drug checking in conjunction with supervised consumption services. However, further benefit may also be afforded by offering such services in community settings where supervised consumption services are not available. In addition, while this study used a relatively simple and inexpensive drug checking technology, additional information may be gained with the use of more advanced drug checking technologies or through the combination of such technologies. Governments and health authorities should work with community partners to further implement and evaluate this potentially important harm reduction intervention.

## Abbreviations

BC: British Columbia
CI: Confidence intervals
FORECAST: Fentanyl Overdose Reduction Checking Analysis Study
OR: Odds ratios
PWUD: People who use drugs
SIF: Supervised injection facility
